# The application of imaging mass cytometry in the immune microenvironment of alveolar echinococcosis

**DOI:** 10.3389/fcimb.2026.1894249

**Published:** 2026-08-24

**Authors:** Dalin Shi, Mingquan Pang, Chengwei Tie, Haining Fan

**Affiliations:** 1 Research Center for High Altitude Medicine, Qinghai Provincial Key Laboratory of Plateau Medical Application, Key Laboratory of Ministry of Education, Qinghai-Utah Joint Research Key Laboratory for High Altitude Medicine, Qinghai University, Xining, Qinghai, China; 2 Qinghai Research Key Laboratory for Echinococcus, Qinghai University, Xining, Qinghai, China; 3 Central Laboratory, Affiliated Hospital of Qinghai University, Xining, Qinghai, China; 4 Department of Hepatopancreatobiliary Surgery, Affiliated Hospital of Qinghai University, Xining, Qinghai, China; 5 Qinghai University School of Clinical Medicine, Qinghai University, Xining, Qinghai, China

**Keywords:** hepatic alveolar echinococcosis, imaging mass cytometry, immune cell, spatial heterogeneity, zoonotic disease

## Abstract

**Background:**

Hepatic alveolar echinococcosis (HAE) is a chronic parasitic disease caused by infection with the larval form of Echinococcus multilocularis, characterised by the formation of invasive lesions in the liver, which involve the proliferation of immune system cells in response to the pathogen. Although surgical resection is the treatment of choice, patients in advanced stages often lose the opportunity for surgery, and drug therapy has limited efficacy. The immune microenvironment plays a key role in tumour progression, but its spatial heterogeneity in HAE has not yet been systematically elucidated.

**Methods:**

Tissue samples for this study were obtained from the tissue proximal to the lesion and matched distal liver tissue from three patients with HAE. Imaging mass cytometry (IMC) analysis was performed using an antibody panel comprising 28 immunological markers. PhenoGraph clustering and t-SNE dimensionality reduction techniques were applied to classify and visualise single-cell data, and to analyse spatial interactions between cells.

**Results:**

Both the tissue proximal to the lesion and the distal liver tissue exhibited significant immune cell infiltration and marked spatial heterogeneity. The tissue proximal to the lesion was characterised primarily by activated CD8^+^ T cells (Cluster 3/19) and CD56^+^ NK cells (Cluster 17/18), presenting an immune-activated state and forming tertiary lymphoid structure (TLS)-like structures. B cells (Cluster 1) were reduced in number in the proximal lesion, whilst macrophages (Cluster 6) showed significant colocalisation with B cells, suggesting that macrophages may regulate B cell function through processes such as antigen presentation.

**Conclusion:**

This study utilised IMC technology to reveal the complex landscape and heterogeneity of the HAE immune microenvironment, providing new targets and insights for the development of immunotherapeutic strategies for this disease.

## 1 Introduction

Hepatic alveolar echinococcosis (HAE) is a fatal zoonotic disease caused by larval infection of *Echinococcus multilocularis*, which exhibits invasive growth and metastatic characteristics driven by the proliferation of immune system cells in response to the pathogen (Bresson-Hadni et al., 2021). The 10-year case fatality rate of untreated HAE patients exceeds 90%, while existing therapies (such as surgical resection and benzimidazole drugs) show extremely limited efficacy due to diffuse lesion distribution and the barrier of fibrotic tissue (Huang et al., 2024; Pavlidis et al., 2025). Although recent studies have revealed the critical role of host immune responses in HAE progression (e.g., dynamic regulation of neutrophils, macrophages, and fibroblasts) (Aji et al., 2018; Jensenius et al., 2024), the spatial heterogeneity of the immune microenvironment and its association with parasite immune escape remain unclear, severely restricting the development of targeted therapeutic strategies.

The interaction between the immune system and parasites plays a central regulatory role in the progression of HAE (Xu et al., 2022). Previous studies have shown that Th1/Th2 immune imbalance, abnormal activation of regulatory T cells (Tregs), and infiltration of myeloid-derived suppressor cells (MDSCs) in the lesion microenvironment collectively constitute an inhibitory immune microenvironment, promoting parasite immune escape and exacerbating liver tissue damage (Liu et al., 2020; Abulizi et al., 2025). However, traditional immunological techniques (such as flow cytometry and immunohistochemistry) have significant limitations: flow cytometry relies on single-cell suspension preparation, losing *in-situ* spatial information of tissues; Immunohistochemistry was constrained by limited detection parameters (typically ≤5 markers), making it difficult to capture phenotypic heterogeneity of immune cells and cell-cell interactions in complex microenvironments. The spatial structural characteristics of tissue microenvironments (such as regional distribution of immune cells and neighborhood associations of cell subsets) are crucial for the initiation and polarization of immune responses, while the “spatial blindness” of traditional techniques severely restricts the systematic understanding of the HAE immune microenvironment (Sussman et al., 2024; Tulading et al., 2025).

Imaging Mass Cytometry (IMC) achieves high-throughput detection of ≥40 cell markers in tissues by conjugating antibodies with metal isotopes. Combined with single-cell resolution spatial localization analysis, it can accurately depict the cell composition, phenotypic characteristics, and spatial interaction networks in complex microenvironments (Baharlou et al., 2019). This technology has shown unique advantages in the study of tumor immune microenvironment, such as revealing the association between immune cell infiltration patterns and cancer prognosis. However, its application in the field of parasitic diseases is still in its infancy (Liu et al., 2022). Scientific questions such as whether there is spatial heterogeneity in the immune microenvironment of different regions within HAE lesions (such as the lesion edge and distal liver tissue of the lesion), and how the positional distribution of key immune cell subsets affects host-parasite interactions, urgently need to be answered through high-dimensional spatial resolution technologies.

Given the advantages of IMC technology in multi-parameter spatial analysis, this study aims to construct an immunocellular atlas of HAE liver tissue and systematically characterise the landscape features of the HAE immune microenvironment. By analysing differences in the composition of immune cells between tissue proximal to the lesion and distal liver tissue in three HAE patients, the study aims to reveal potential associations between spatial heterogeneity and disease progression.

## 2 Materials and methods

### 2.1 Case information

Case 1: Female, aged 33. Medical history: Long-term residence in the Qinghai region; history of drinking untreated water. Clinical presentation: The patient was admitted to hospital due to dull pain in the right upper abdomen accompanied by weight loss over a period of two months. CT scan results revealed an irregular, infiltrative hypodense lesion in the right lobe of the liver with pseudocystic margins; a diagnosis of HAE was considered. A liver biopsy was performed, and the pathological diagnosis confirmed a multi-chambered Echinococcus cystic hydatid disease.

Case 2: Male, aged 25, a herdsman. Admitted for “right upper abdominal pain of over three months’ duration, worsening over the past 10 days, accompanied by jaundice, with no apparent cause”. The patient first experienced abdominal pain three months prior but received no specific treatment; he sought medical attention after noticing an increase in pain and the onset of jaundice over the past 10 days. The patient presented with jaundice, a positive Murphy’s sign, tenderness in the gallbladder region, and the liver and gallbladder were not palpable subcostally. A liver biopsy was performed, and the pathological diagnosis was a multilocular echinococcal cyst.

Case 3: Female, 45 years old, herdswoman. Admitted for right upper abdominal pain of two months’ duration with no apparent cause, accompanied by jaundice. CT scan results revealed an irregular, infiltrative hypodense lesion in the left lobe of the liver with pseudocystic margins, suggestive of HAE. A liver biopsy was performed, and histopathological examination confirmed a multilocular echinococcal cyst.

This study was approved by the Ethics Committee of Qinghai University Affiliated Hospital, and all patients provided written informed consent. This study was approved by the Ethics Committee of Qinghai University Affiliated Hospital, and all patients provided written informed consent. Detailed clinical characteristics, including individual hepatic biochemical parameters and peripheral blood leukocyte profiles, are shown in **Table 1**. Regarding prior pharmacotherapy, Patient 1 received albendazole for 84 months prior to surgery, whereas Patients 2 and 3 had no recorded history of prior albendazole therapy before admission. The inclusion of these systemic parameters allows for a clearer distinction between the local tissue immune microenvironment and the systemic immune state, providing context for the correlation between tissue findings and clinical severity.

**Table 1:** Imaging mass cytometry HAE patients information.

Patients ID	Sex	Age (years)	PNM stage	Lesion max diameter(cm)	Lesion site	Hepatic biochemical parameters	Peripheral blood leukocyteS
HAE 1	F	33	P4N1M0	16.0	Distal lesion	ALT: 32 U/L AST: 31 U/L ALP: 31 U/L GGT: 50 U/L TBIL: 73.9 μmol/L ALB: 33.4 g/L	WBC: 3.04 ×10^9^/L NEU: 2.31 ×10^9^/L LYM: 0.46 ×10^9^/L MON: 0.15 ×10^9^/L EOS: 0.10 ×10^9^/L
					Proximal lesion		
HAE 2	M	25	P4N1M1	17.0	Distal lesion	ALT: 151 U/L AST: 159 U/L ALP: 133 U/L GGT: 52 U/L TBIL: 10.2 μmol/L ALB: 39.4 g/L	WBC: 8.85 ×10^9^/L NEU: 5.80 ×10^9^/L LYM: 1.87 ×10^9^/L MON: 1.00 ×10^9^/L EOS: 0.15 ×10^9^/L
					Proximal lesion		
HAE 3	F	45	P3N1M0	13.0	Distal lesion	ALT: 37 U/L AST: 27 U/L ALP: 113 U/L GGT: 23 U/L TBIL: 9.2 μmol/L ALB: 41.3 g/L	WBC: 6.55 ×10^9^/L NEU: 4.04 ×10^9^/L LYM: 1.10 ×10^9^/L MON: 0.45 ×10^9^/L EOS: 0.88 ×10^9^/L
					Proximal lesion		

### 2.2 Sample collection and preparation

In accordance with the diagnostic criteria set out in the ‘ Expert Consensus on the Imaging Diagnosis of Hepatic Echinococcosis’ (Liu et al., 2021), tissue samples were collected from the region adjacent to the lesion in three patients. Paired distal liver tissue samples (at least 2 cm from the edge of the lesion) were also collected for comparison. All samples were fixed in 10% neutral buffered formalin, embedded in paraffin, and sectioned into 5 μm-thick serial sections. Following dewaxing and rehydration, the sections underwent heat-induced epitope retrieval (HIER) using a high-pressure heating method (with pH 9.0 Tris-EDTA buffer, 121 °C, 15 psi). After cooling, the sections were rinsed three times with phosphate-buffered saline (PBS) for 5 minutes each time.

### 2.3 Preparation of the IMC antibody panel

Based on the IMC principle, a detection panel comprising 28 antibodies was systematically designed. This panel was constructed by precisely matching antibodies targeting lineage-specific markers and functional molecules of liver immune cell subsets with unique, identifiable metal isotope labels. This panel covers markers for lymphocytes (B cells, T cells, dendritic cells, tissue macrophages), hepatic cell proliferation, cell activation, and molecules associated with immunotherapy (**Table 2**).

**Table 2:** HAE antibody panel for imaging mass cytometry.

Cell category	Specificity	Purpose	Clone	Lable
Immune cells	CD45	pan-immune cells	D9M8I	Dy163
lymphocyte	B cells			
	CD19	B cells	6OMP31	Nd142
	T cells			
	CD3	T and NKT cells	Polyclonal, C-Termina	Er170
	CD4	CD4+ T cells	EPR6855	Gd156
	CD8a	CD8+ T cells	C8/144B	Dy162
	Foxp3	Tregs cells	206D	Gd155
	T-bet	Lineage-specific transcription factor of Th1 cell subset	4B10	Lu175
	Gata-3	Lineage-specific transcription factor of Th2 cell subset	EPR16651	Yb176
	BCL-6	Lineage-specific transcription factor of Tfh cell subset	KA4B18	Er167
	IL-17a	Interleukin-17	BL168	Gd158
	CD45RA	Naïve T cells	MEM-56	Nd143
	CD45RO	Memory T cells	UCH-L1	Er166
	Myeloid-derived cells			
	CD11c	Dendritic cells	EP1347Y	Sm154
	CD68	Macrophages	KP1	Tb159
	CD15	Granulocytes	HI98	Eu153
	CD56	NK and NKT cells	RNL-1	Sm152
	CD33	Myeloid cells	WM53	Nd145
	Hepatic stellate cells			
	α-SMA	Myofibroblast markers	1A4	Pr141
	TGF-β	Transforming Growth Factor -β	TW4-2H10	Eu151
Hepatocyte proliferation	Ki-67	Proliferation marker	B56	Er168
	Collagen I	Type I Collagen	COL-1	Tm169
Cell activation	IFN-γ	Interferon-γ	4S.B3	Sm147
	TNF-α	Tumor Necrosis Factor-α	MAb11	Gd160
	IL-6	Interleukin - 6	MQ2-13A5	Nd148
	HLA-DR	Immune activation	YE2/36 HLK	Yb174
PD-1 immunotherapy	CD274	PD-L1	E1L3N	Nd150
	CD273	PD-L2	176611	Yb172
	CD279	Programmed Cell Death Protein 1 (PD-1)	EPR4877(2)	Ho165

### 2.4 Hyperion tissue imager scanning

Image acquisition was performed using the Hyperion™ high-resolution imaging system (Fluidigm). During the procedure, tissue sections were subjected to laser ablation via a raster scan at a frequency of 200 Hz, with the target area being the largest possible square region within each sample. Pre-processing of raw data and experimental quality validation were performed using the manufacturer-provided MCD Viewer™ image analysis software (Fluidigm), which was also utilised to generate selective multi-channel composite images of the markers. Following quality validation, the data were pre-processed and exported in a standardised format to serve as the baseline dataset for subsequent in-depth analysis (**Supplementary Figure 1A**). Cell segmentation quality control: cell nuclei were positioned at the centre of the segmentation mask, and cell typing conformed to established criteria (**Supplementary Figure 1B**). Cell size quality control: cells with an area greater than 50 µm² were included in the analysis, confirming that cell size quality control was satisfactory (**Supplementary Figure 1C**).

### 2.5 IMC data processing

Raw.mcd files were converted to TIFF format using the imctools toolkit (https://github.com/BodenmillerGroup/imctools). Single-cell segmentation was performed using CellProfiler software (version 3.1.8). Cell nuclei were identified using Fluidigm DNA embedding agents (Ir191/Ir193), whilst cell membranes were detected using a panel of markers including CD3, CD8, CD19, CD56, pan-cytokeratin (pan-CK) and α-smooth muscle actin (α-SMA). Subsequently, single-cell object masks were generated using CellProfiler, producing TIFF files containing the spatial coordinates and boundaries of the cells.

Outliers were removed from the single-cell data by setting a 95th percentile threshold. Inter-sample normalisation was performed using the Harmony algorithm to correct for technical errors caused by staining batch and instrument variation. The normalised data were further converted to Z-scores to enable comparability between different markers, thereby facilitating subsequent clustering and visualisation analyses.

### 2.6 Cluster analysis and barnes-hut t-SNE dimension reduction

Cell phenotypes were clustered using the PhenoGraph algorithm. This method calculates inter-cell similarity based on Euclidean distance and optimises community partitioning via the Louvain algorithm to classify cells into distinct subpopulations (e.g., T-cell subpopulations, myeloid cell subpopulations). Data normalised using Harmony were subjected to dimensionality reduction via the Barnes-Hut t-SNE algorithm (confusion index = 42, default parameters). This step maps high-dimensional features onto a two-dimensional space to visually illustrate the spatial distribution of cell subpopulations.

### 2.7 Statistical analysis

In the analysis of spatial interactions between cells, permutation tests were employed to assess whether the observed colocalisation relationships between each pair of cell types deviated significantly from a random distribution. For each image, the spatial positions of the cells were randomly permuted 1, 000 times to construct a null distribution, and the probability that the observed frequency exceeded the random expectation was calculated. All statistical analyses in this study were performed using the R software. The normality of the data distribution for the continuous variables was assessed using the Shapiro-Wilk test. Given the extremely small sample size, non-parametric tests (specifically the Wilcoxon signed-rank test for paired comparisons of cell densities between proximal and distal lesions) were predominantly applied to ensure statistical validity. The main R packages used included: imcRtools for spatial interaction analysis and cell neighbourhood construction; ggplot2 and ComplexHeatmap for data visualisation. A p-value of <0.05 was considered statistically significant.

## 3 Results

### 3.1 The immune microenvironment landscape of the lesion and surrounding tissues

**Figure 1:**
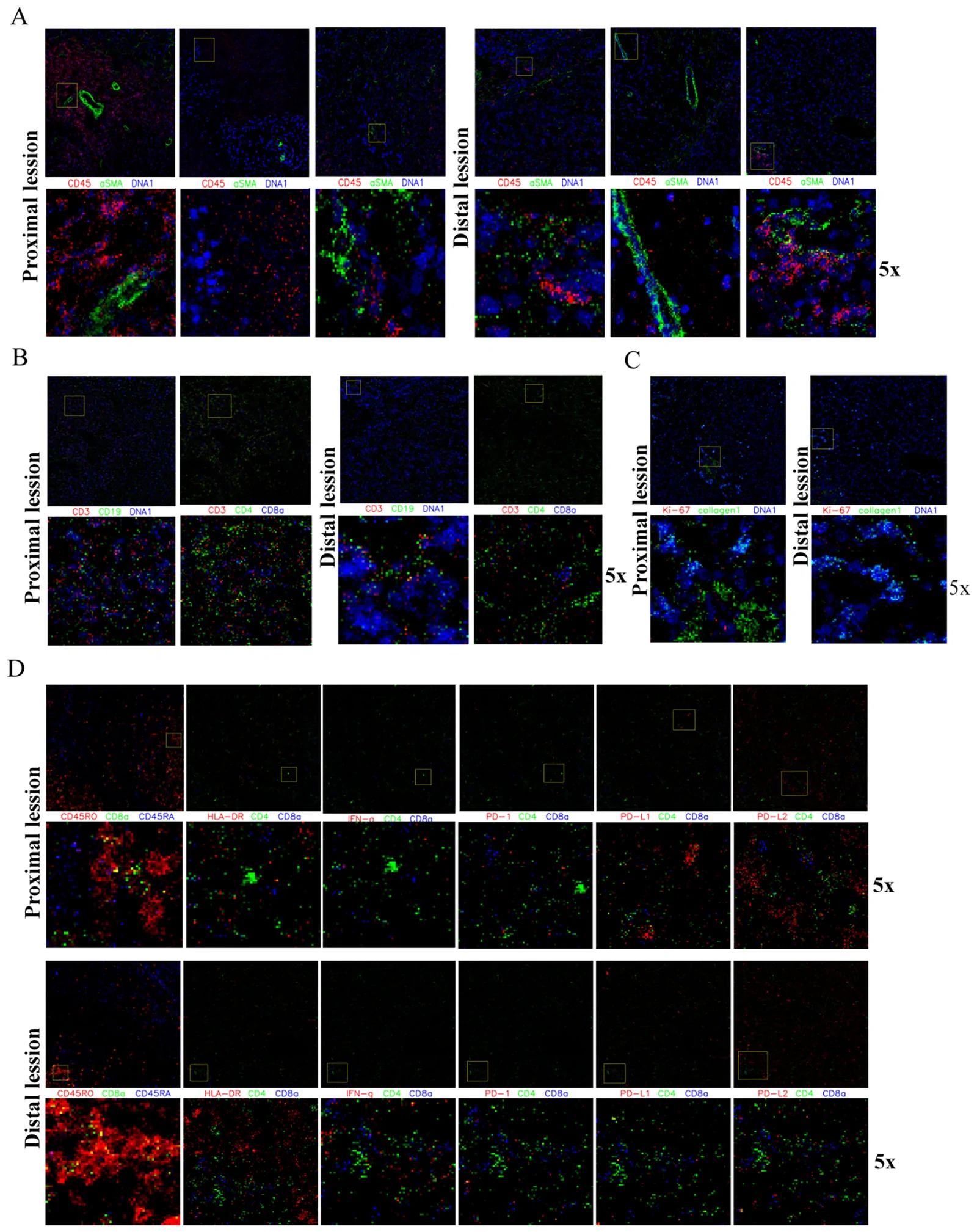
Spatial profiling of immune cell distribution and activation markers in proximal and distal lesions. **(A)** Pan-immune cell markers. **(B)** T/B lymphocyte markers. **(C)** Cellular proliferation markers. **(D)** CD8^+^ T cell activation markers.

**Figure 2:**
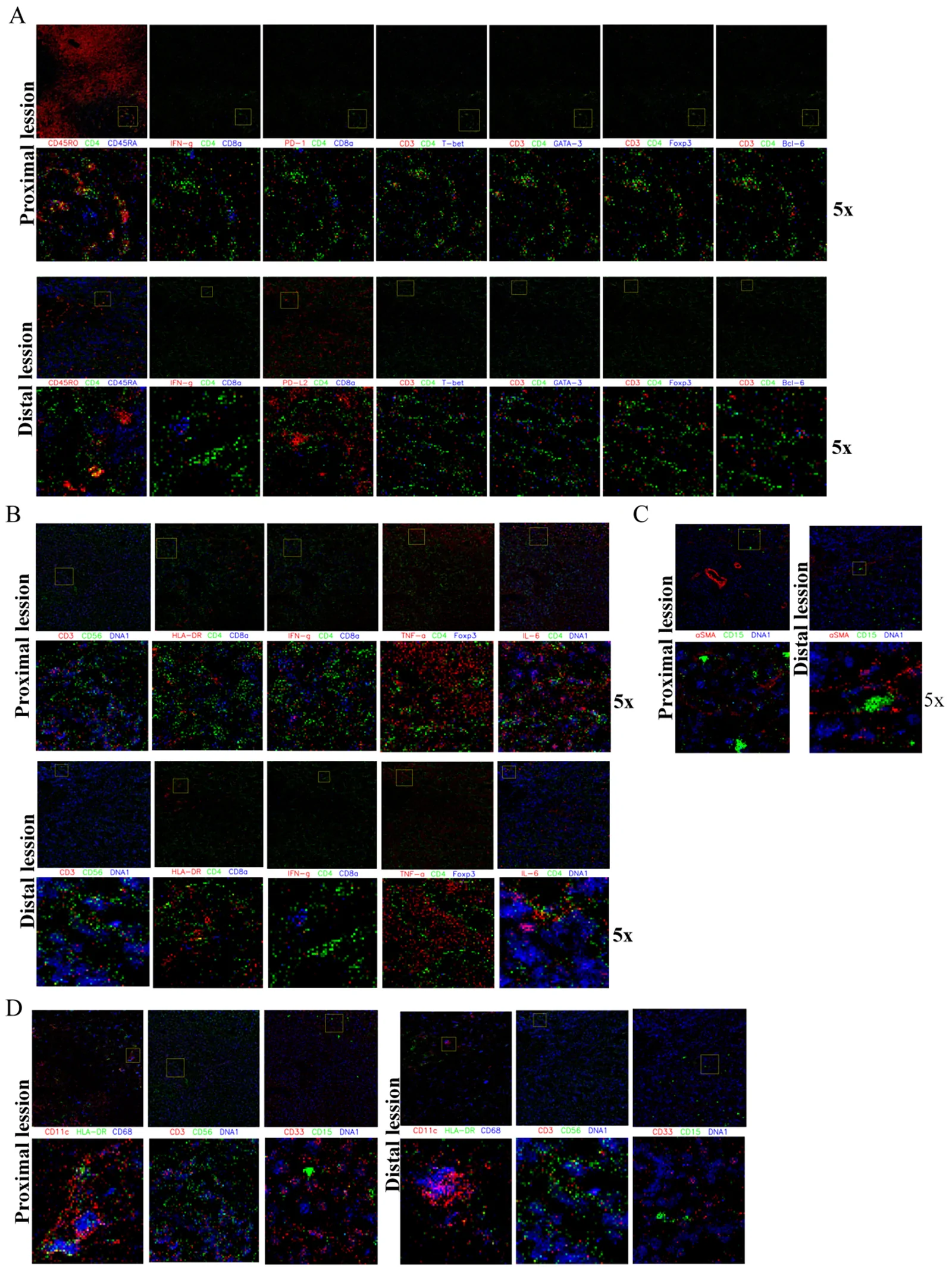
Subset-specific spatial mapping of immune and structural markers. **(A)** CD4^+^ T cell activation markers. **(B)** NK/NKT cell markers. **(C)** Structural protein expression. **(D)** Myeloid cell markers.

The results showed significant immune cell infiltration in both the marginal and distal regions of the lesion (**Figures 1** and **2**), indicating that the immune composition of the two regions was broadly similar. Abundant CD45-positive immune cells infiltrated both the margins and distal regions of the lesions, with large populations of myeloid and lymphoid cell subsets. Notably, these immune cells exhibited a spatially clustered distribution pattern, forming tissues with characteristics of tertiary lymphoid structures (TLS) (**Figure 1A**).

#### 3.1.1 Functional heterogeneity of T-lymphocyte subpopulations

CD3^+^ T cells constituted the major component of the infiltrating lymphocytes and exhibited significant spatial heterogeneity (**Figure 1B**).

CD8^+^ T-cell phenotype: Both the proximal and distal regions of the lesion displayed CD8^+^ T-cell populations predominantly comprising CD45RO^+^ memory cells, but their functional states were markedly different. Proximal to the lesion: These cells exhibit a quiescent phenotype (HLA-DR^-^, IFN-γ^-^, PD-1^-^), and no colocalisation of PD-L1/PD-L2 was observed. Distal to the lesion: These cells exhibit an activated phenotype (HLA-DR^+^, IFN-γ^+^, PD-1^+^) (**Figure 1D**).

CD4^+^ T-cell phenotype: CD4^+^ T cells in the proximal region of the lesion exhibit an effector-like phenotype (CD45RA^+^, TNF-α^+^, IFN-γ^+^, PD-1^+^), but the expression of key transcription factors (T-bet, GATA-3, Foxp3, Bcl-6) is reduced. This characteristic suggests that they represent incompletely differentiated early helper T cells (**Figure 2A**).

#### 3.1.2 Interactions between regulatory T cells and Th subpopulations

Foxp3^+^ regulatory T cells (Tregs) within the lesion area exhibited elevated TGF-β expression, indicating that they were in a functionally activated state. Spatial colocalisation analysis revealed significant spatial exclusion between Tregs and Th1 cells. Furthermore, heterogeneous helper T cell subsets were detected within the microenvironment, including Th2 cells (GATA-3^+^), follicular helper T cells (Tfh, Bcl-6^+^) and Th17 cells (IL-17^+^) (**Figure 2A**).

#### 3.1.3 Activation characteristics of innate immune cells

NK/NKT cells (CD56^+^) within the perilesional area exhibited a highly activated phenotype (HLA-DR^+^IFN-γ^+^), accompanied by elevated expression of TNF-α and IL-6, indicating a state of intense activation (**Figure 2B**). Among myeloid cells, the monocyte/macrophage population exhibited upregulation of HLA-DR, TNF-α and IFN-γ expression, suggesting enhanced antigen-presenting capacity and pro-inflammatory activity (**Figure 2D**). Furthermore, the density of B cells (CD19^+^) in the proximal region of the lesion was significantly lower than in the distal region.

#### 3.1.4 Dynamic characteristics of the immune microenvironment

Proliferation analysis revealed the presence of Ki-67^+^ follicular helper T (Tfh) cells in both the proximal and distal regions of the lesion, suggesting active proliferative activity. Regarding vascular distribution, α-SMA staining confirmed the presence of a dense vascular network in these regions, which may provide a structural framework for sustained immune cell infiltration (**Figure 2C**).

### 3.2 Analysis of the heterogeneity of the HAE immune microenvironment

#### 3.2.1 Differential protein expression profiles

Compared with the distal lesions, the proximal lesions exhibited significantly higher expression of immune markers, displaying two characteristic expression patterns (**Supplementary Figures 2A, B**): Imbalance in the Immune Activation-Suppression Balance: The proximal region of the lesion specifically enriched for IFN-γ^+^, IL-6^+^, PD-L1^+^ and CD33^+^ cell populations (with the first three being particularly prominent). This indicates that intense immune activation coexists with compensatory PD-L1/CD33-mediated immune suppression mechanisms to maintain immune homeostasis. Activation of immune effector pathways: Expression of CD15^+^ (a neutrophil marker), CD8a^+^ and IL-17a^+^ was generally elevated, but did not form new cell subsets. This reflects the infiltration of CD8^+^ T cells, the recruitment of neutrophils and the activation of the Th17 pathway, which collectively constitute a locally active immune microenvironment.

#### 3.2.2 Cell cluster analysis

**Figure 3:**
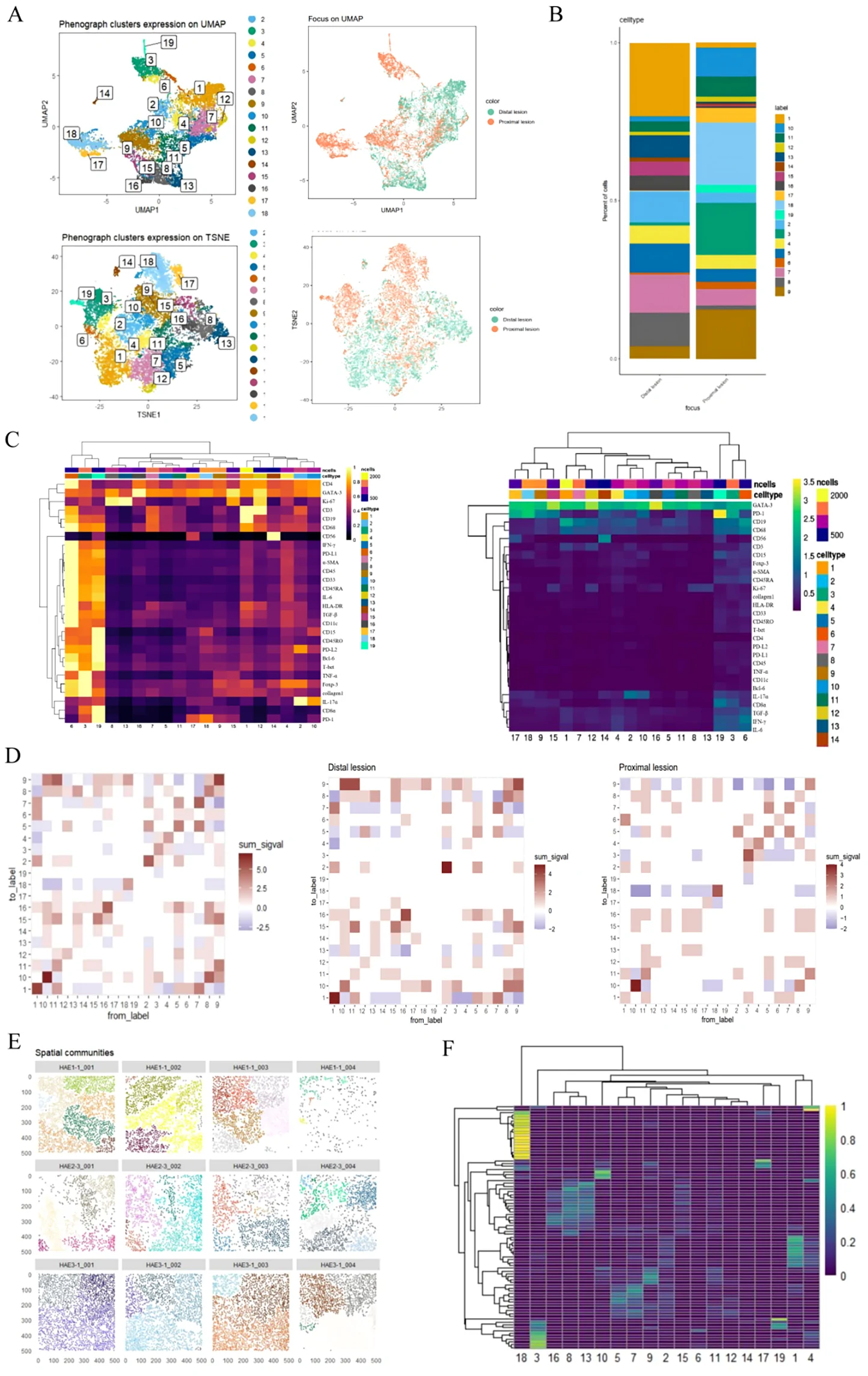
Cellular clustering and spatial analysis via imaging mass cytometry. **(A)** PhenoGraph clustering visualizations (Top: UMAP projection;Bottom: t-SNE projection). **(B)** Proportional distribution of cellular clusters across lesion types. **(C)** Clustering heatmap of antibody expression across cellular clusters (left panel: z-score normalized dat; X-axis = cellular clusters; Y-axis = antibodies; Color scale = antibody expression intensity; n(cells) = cell count per cluster). **(D)** Inter-cluster correlation networks (left to right): Global - Distal lesions - Proximal lesions. **(E)** Spatial mapping of cellular clusters across tissue sections. **(F)** Heatmap illustrating the correlation of interactions between cell subpopulations based on imaging mass cytometry data. The rows and columns correspond to different PhenoGraph cell subpopulations, respectively, whilst the colour scale represents the correlation coefficient of interactions between cell subpopulations, ranging from 0 to 1; a higher value indicates a stronger correlation between the interactions of the two subpopulations.

Through dimensionality reduction using UMAP and t-SNE combined with PhenoGraph clustering, 19 distinct cell clusters were identified (**Supplementary Figure 2B**; **Figure 3A**). Spatial heterogeneity is characterised as follows:

Lesion-proximal specific clusters: Clusters 17 and 18 were significantly enriched in tissue proximal to the lesion compared with distal tissue (with a marked increase in the proportion of Cluster 18). Both clusters exhibited high PD-1 expression. Cluster 17 displayed low levels of CD56 (an NK cell marker) expression, suggesting NK cell infiltration and functional activation. Furthermore, Clusters 3 and 19 (predominantly Cluster 3), which are mainly localised at the lesion margin, exhibited elevated expression of PD-1, CD8, IFN-γ and IL-6, and were identified as activated CD8^+^ T-cell subsets (**Supplementary Figure 2A**; **Figures 3B, C**). This indicates the presence of specific CD8^+^ T-cell infiltration and functional activation in the proximal region of the lesion.

Clusters enriched in the proximal region of the lesion: Clusters 9, 10 and 11 exhibited relatively high proportions in the proximal region of the lesion. Although cluster 11 exhibited high IL-17α expression, comparable IL-17α^+^ expression in distal cluster 2 and the low abundance of cluster 2 at the lesion margin resulted in no significant difference in overall IL-17α levels between regions.

Clusters specific to the distal region of the lesion: Clusters 1 (CD19^+^ B cells) and 16 (GATA-3^+^ Th2 cells) showed a significantly increased proportion in the distal region (p<0.001), indicating an accumulation of B cells and Th2 cells. Clusters 8, 13 and 15 exhibited high Ki-67 expression (a proliferation marker) but lacked typical immune markers, and were designated as proliferative non-immune cells. Furthermore, clusters 2 (IL-17a^+^), 5 and 7 showed a preference for distribution in the distal region. In summary, the distal region of the lesion is characterised by a specific enrichment of B cells (Cluster 1), whereas the proximal region is characterised by significant infiltration of activated CD8^+^ T cells (Clusters 3/19) and NK cells (Clusters 17/18).

### 3.3 Characteristics of spatial interaction networks

#### 3.3.1 Cell distribution properties

Clusters 14 and 19 exhibited weak spatial autocorrelation, indicating a diffuse distribution within the tissue. Cluster 1 (B cells) and Cluster 7 (macrophages) exhibit strong spatial co-localisation and high co-expression of CD3/CD19/CD68, suggesting the possible presence of homologous cell populations or shared differentiation pathways. Cluster 9 (CD4^+^ T cells) interacts strongly with multiple clusters, indicating that it plays a central hub role in the immune microenvironment (**Figures 3D, E**).

#### 3.3.2 Differences in region-specific interactions

Enhanced interactions proximal to the lesion: Significant colocalisation was observed between Cluster 1 (B cells) and Cluster 6 (activated macrophages), suggesting that macrophages may mediate phagocytosis or antigen presentation to B cells. Enhanced interactions between Cluster 9 (follicular helper T cells, Tfh) and Cluster 1 (B cells) provide a cellular basis for antibody production within the TLS (**Figure 3D**).

Distal enhanced interactions: A significant interaction exists between cluster 2 (Th17 cells) and cluster 5 (neutrophils), which may drive an IL-17α-mediated inflammatory cascade, thereby promoting tissue fibrosis (**Figure 3D**).

## 4 Discussion

### 4.1 Functional polarisation of immune cell infiltration

T lymphocytes constitute the primary population of infiltrating lymphocytes, and their distribution exhibits significant spatial heterogeneity. CD8^+^ T cells in the proximal regions of the lesion remain in a resting state, whereas their distal counterparts exhibit activated characteristics (Wang H. et al., 2017). This spatial dichotomy in the functional state of CD8^+^ T cells (proximal quiescence vs distal activation) reflects the dynamic microenvironmental regulation of the immune response (Zhao et al., 2023). This functional polarisation may arise from differences in the gradient of parasite antigen exposure and the distribution of matrix-derived factors across distinct spatial niches, driven by regional variations in immune pressure and the cytokine environment (Zhang et al., 2020). Specifically, the prominent enrichment of PD-1-expressing exhausted CD8^+^ T cells (Clusters 3 and 19) in the proximal regions provides critical spatial validation for the T-cell exhaustion models previously characterized in chronic alveolar echinococcosis (Yang et al., 2024). The prolonged presence of parasitic antigens in this micro-niche drives this exhaustion to facilitate immune tolerance.

In the regions proximal to the lesion, CD56^+^ NK cells (HLA-DR^+^ IFN-γ^+^ TNF-α^+^) colocalise with functionally silenced CD8^+^ T cells (CD45RO^+^HLA-DR^-^PD-1^-^), revealing a unique mechanism of immune evasion. Although NK cells maintain frontline defence by directly killing the parasite, the functional suppression of CD8^+^ T cells (manifested by the absence of PD-L1/PD-L2 co-stimulation and metabolic quiescence markers) prevents tissue damage caused by excessive cytotoxic activation (Hübner et al., 2006; Wang J. et al., 2017; Wu et al., 2021). This mechanism of spatial immune compartmentalisation parallels the ‘divided tolerance’ phenomenon observed in chronic viral infections, achieving a homeostatic balance between pathogen control and host survival through selective immune suppression.

It is worth noting that the arrest of CD4^+^ T-cell differentiation (T-bet^low^/GATA-3^low^), coupled with excessive Treg activation, jointly establishes an immunoprivilege microenvironment conducive to the long-term survival of the parasite (Li et al., 2023; Wang et al., 2024). This finding is consistent with immune regulatory strategies observed in chronic helminth infections, wherein coordinated Th2 polarisation and Treg expansion drive host-parasite immune compromise (Zhang et al., 2016; Lin et al., 2024). The spatial immunodynamics revealed here share fundamental principles with the adaptive evolution of the cystic stage of cysticercosis—specifically, the transition from neutrophil-driven early inflammation to fibrotic encapsulation—highlighting the conservation of this host defence strategy across phylogenetically diverse parasites.

### 4.2 Compartmental variations in the immune microenvironment

The immune microenvironment in HAE exhibits significant spatial heterogeneity. Compared with distal tissues, the expression of immune markers is markedly elevated in the regions proximal to the lesion, reflecting a characteristic imbalance in the immune activation-suppression equilibrium and the activation of immune effector pathways (Lu et al., 2025; Ren et al., 2025). The intense immune activation in the proximal region induces compensatory PD-L1/CD33-mediated immune suppression, a potential physiological adaptation designed to prevent immunopathological damage, yet simultaneously creating opportunities for pathogen immune evasion (Guo et al., 2026).

Cluster analysis further delineates the compartment-specific distribution of immune cells: the region proximal to the lesion is characterised by significant infiltration of activated CD8^+^ T cells and NK cells, whereas distal tissues exhibit an enrichment of B cells and Th2 cells (Vignali et al., 2023). This compartmentalised cellular architecture may correspond to distinct immune requirements and microenvironmental factors (Ouyang et al., 2025). For example, the proximal region may require robust cell-mediated immunity to directly clear pathogens, whereas the distal compartments may maintain immune homeostasis through humoral immunity and regulatory responses (Ran et al., 2026).

### 4.3 The complexity of cellular interaction networks

Analysis of spatial interaction networks reveals significant differences in the distribution patterns and interactions among different cell populations within tissues. CD4^+^ T cells exhibit strong interactions with multiple cell populations, suggesting that they play a central role in the immune microenvironment by regulating cell activation, proliferation and differentiation through interactions with other immune cells (Bellanger et al., 2021). Macrophages (Cluster 6) and B cells (Cluster 1) show significant colocalisation in the proximal regions, although the density of B cells in the proximal regions is significantly lower than in the distal regions. This phenomenon suggests that macrophages may participate in B-cell clearance or antigen presentation via phagocytosis, although the specific mechanisms require further investigation (Bektan Kanat et al., 2024). Given that chronic Echinococcus multilocularis infection frequently exploits hepatic Kupffer cells and recruited macrophages to foster a tolerogenic and immunosuppressive microenvironment (Wang et al., 2009), this spatial proximity suggests that macrophages may not only engage in antigen presentation but actively suppress B cell proliferation or mediate localized immune clearance to sustain peripheral tolerance.The enhanced interaction between follicular helper T cells (Cluster 9) and B cells provides a cellular basis for antibody production and the regulation of immune responses. Furthermore, the significant spatial interactions between Th17 cells (Cluster 2) and neutrophils (Cluster 5) in the distal region strongly mirror the inflammatory cascades typically associated with helminth-induced tissue fibrosis, confirming a distinct spatial compartmentalization where proximal tolerance functionally contrasts with distal inflammation (Mourglia-Ettlin et al., 2018). These complex and intricate networks of cell-cell interactions exert a significant influence on the disease progression and outcomes of HAE.

### 4.4 Limitations and future directions

This study has certain limitations. Firstly, the sample size is relatively small (comprising paired tissue samples from only three patients), and there is a lack of samples from different stages of the disease course (such as early versus late stages), which may limit the generalisability of the study’s conclusions. Furthermore, individual factors, such as the significant age differences among our patients, may lead to distinct baseline immunological profiles. Due to the limited sample size, we were unable to perform separate statistical descriptions and subset analyses for each clinical case. Additionally, the use of prior pharmacotherapy in our cohort, notably the long-term albendazole treatment in Patient 1, introduces documented immunomodulatory effects that could confound the interpretation of the observed immune activation and exhaustion profiles (Wu et al., 2021). Furthermore, the lack of matched systemic serum cytokine data limits our ability to fully correlate local tissue microenvironment characteristics with the systemic immune state.Consequently, the current findings should be interpreted as hypothesis-generating observations rather than consolidated evidence. Secondly, as the study is primarily based on cross-sectional data, it can only reflect the immune status at a specific point in time, making it difficult to reveal the dynamic changes in the immune microenvironment as the disease progresses. Future work will involve multi-centre, large-scale cohort studies incorporating HAE patients at different clinical stages to verify whether the immune characteristics identified in this study have broad clinical relevance.

## 5 Conclusion

This study employs IMC to delineate the complex landscape and profound spatial heterogeneity within the HAE immune microenvironment. Significant immune cell infiltration exhibiting marked spatial heterogeneity was observed in both proximal lesional and distal tissues. Proximal lesion demonstrated reduced B cell abundance alongside significant B cell-macrophage co-localization, suggesting macrophage involvement in B cell functional regulation via antigen presentation and related mechanisms. Concurrently, cellular proliferation was notably suppressed in proximal ilesion. These findings deepen our mechanistic understanding of HAE immunopathogenesis—particularly its microenvironmental heterogeneity and cellular interaction networks—providing novel potential targets and conceptual frameworks for developing immunotherapeutic strategies against this disease.

## Data Availability

The original contributions presented in the study are included in the article/**Supplementary Material**. Further inquiries can be directed to the corresponding author.

## Supplementary material

Supplementary Figure 1 Hyperion mass cytometry scanning and quality control. **(A)** Hyperion mass cytometry scanning flowchart. **(B)** Hyperion mass spectrometry imaging. Note: Nuclei are centered within the segmentation template - cell types meet standards. **(C)** Boxplot of cell size across different lesions. *Note: The X-axis represents different lesions, the Y-axis represents cell area (measured by area); Quality control passes as cell area > 50 meets the requirement.

Supplementary Figure 2 Differential protein expression analysis in perilesional and distal tissues. **(A)** Marker expression ridge plots across lesion zones(X-axis = Marker (antibody) expression intensity; Y-axis = Density distribution). **(B)** Violin plots depicting antibody expression profiles across cellular clusters.
